# Impact of Value Frameworks on the Magnitude of Clinical Benefit: Evaluating a Decade of Randomized Trials for Systemic Therapy in Solid Malignancies

**DOI:** 10.3390/curroncol28060412

**Published:** 2021-11-21

**Authors:** Ellen Cusano, Chelsea Wong, Eddy Taguedong, Marcus Vaska, Tasnima Abedin, Nancy Nixon, Safiya Karim, Patricia Tang, Daniel Y. C. Heng, Doreen Ezeife

**Affiliations:** 1 Cumming School of Medicine, University of Calgary, Calgary, AB T2N 1N4, Canada; 2 Faculty of Science, University of Calgary, Calgary, AB T2N 1N4, Canada; chelsea.wong@ucalgary.ca; 3 Faculty of Medicine and Health Sciences, McGill University, Montreal, QC H3A 0G4, Canada; edouarda.taguedong@mail.mcgill.ca; 4 Tom Baker Cancer Centre, Calgary, AB T2N 4N2, Canada; Marcus.Vaska@albertahealthservices.ca (M.V.); Tasnima.Abedin@albertahealthservices.ca (T.A.); Nancy.A.Nixon@albertahealthservices.ca (N.N.); Safiya.Karim@albertahealthservices.ca (S.K.); Patricia.Tang@albertahealthservices.ca (P.T.); Daniel.Heng@albertahealthservices.ca (D.Y.C.H.); Doreen.Ezeife@albertahealthservices.ca (D.E.)

**Keywords:** clinical benefit, clinical trials, value frameworks

## Abstract

In the era of rapid development of new, expensive cancer therapies, value frameworks have been developed to quantify clinical benefit (CB). We assessed the evolution of CB since the 2015 introduction of The American Society of Clinical Oncology and The European Society of Medical Oncology value frameworks. Randomized clinical trials (RCTs) assessing systemic therapies for solid malignancies from 2010 to 2020 were evaluated and CB (Δ) in 2010–2014 (pre-value frameworks (PRE)) were compared to 2015–2020 (POST) for overall survival (OS), progression-free survival (PFS), response rate (RR), and quality of life (QoL). In the 485 studies analyzed (12% PRE and 88% POST), the most common primary endpoint was PFS (49%), followed by OS (20%), RR (12%), and QoL (6%), with a significant increase in OS and decrease in RR as primary endpoints in the POST era (*p* = 0.011). Multivariable analyses revealed significant improvement in ΔOS POST (OR 2.86, 95% CI 0.46 to 5.26, *p* = 0.02) while controlling for other variables. After the development of value frameworks, median ΔOS improved minimally. The impact of value frameworks has yet to be fully realized in RCTs. Efforts to include endpoints shown to impact value, such as QoL, into clinical trials are warranted.

## 1. Introduction

In cancer care, the value of a treatment can be defined as the magnitude of clinical benefit achieved per dollar spent [1]. Increasingly, advances in cancer care are accompanied with rapidly escalating drug prices, with cancer costs rising faster than the gross domestic product per capita in many countries [2]. The magnitude of clinical benefit of new cancer medicines has failed to rise proportionately with the costs, which indicates that the pricing may not indicate the clinical value of these cancer medicines [3]. The oncology community has been striving to promote value-based cancer care in efforts to decrease costs and manage limited resources.

Since 2015, several groups have developed value frameworks that assess the magnitude of clinical benefit while balancing toxicity profile and costs. The American Society of Clinical Oncology (ASCO) and the European Society for Medical Oncology (ESMO) value frameworks consider survival endpoints, in addition to quality of life (QOL) [4,5,6,7]. Establishment of value considerations can ideally inform not only value-based cancer care, but also value-based research. For example, palliative treatments that result in a survival gain of three or more months over the standard-of-care, or improve QOL, are scored highly using the ESMO framework. Thus, one may hypothesize that greater recognition of high-value care may result in more clinical trials demonstrating greater survival gains or QOL improvement. It is unknown whether the introduction of these value frameworks has impacted clinical endpoints in studies published after their development.

In this study, we examined the solid tumour randomized clinical trials published five years before and after the 2015 introduction of the ASCO and ESMO value frameworks. We sought to assess how clinical trial endpoints and the magnitude of clinical benefits have been impacted by knowledge of the importance of value considerations.

## 2. Materials and Methods

### 2.1. Search Strategy and Study Selection

A systematic search for pertinent articles was performed in MEDLINE (Ovid), Evidence-Based Medicine (EBM Reviews), PubMed, CINAHL, MEDLINE (Ebsco), and EMBASE. The search strategy is available in an Appendix A (Table A1 and Table A2). English phase II or III randomized control trials published between 1 January 2010 and 30 November 2020 that evaluated systemic therapies for solid malignancies were included. Randomized phase II trials were included in order to capture drugs treating uncommon tumor types or molecular subtypes, where the feasibility of a randomized phase III trial is poor. All titles and abstracts (full text if required) were reviewed and screened against the inclusion and exclusion criteria. Clinical trials in progress and single-arm studies were excluded. Studies involving radiation monotherapy, treatment for symptoms only, subgroup analyses, reviews of multiple studies or pooled analyses, and protocols were excluded.

### 2.2. Data Abstraction

For each eligible trial, a pair of two independent reviewers (ET and CW) collected information on trial characteristics (title, first author, journal name, year of publication, trial design, type of cancer, disease setting (curative vs. palliative), primary and secondary endpoints), interventions (experimental drug regimen, control regimen, drug mechanism of action, line of therapy), and outcomes of interest (overall survival, progression-free survival, response rate, quality of life). Absolute values for the outcomes were extracted, as well as the associated hazard ratios and 95% confidence intervals, where applicable. A third reviewer (EC) repeated abstraction for a random sample of 50% of the eligible trials to ensure quality. Discrepancies were resolved by consensus or with the assistance of an additional reviewer (DAE) when necessary.

### 2.3. Data Synthesis and Analysis

For each clinical trial, the magnitude of clinical benefit (ΔOS, ΔPFS, ΔRR) was calculated by subtracting the median survival of the control arm (in months) from the median survival of the experimental arm. The data was divided into two groups based on year of publication: 2010–2014 (PRE) and 2015–2020 (POST). The date of publication of the ASCO and ESMO value frameworks in 2015 was used as the cut point. If a trial had greater than one experimental arm, each experimental arm was counted as a separate study.

Statistical analyses compared variables in the PRE and POST time periods. Univariable analyses were performed to compare baseline characteristics between the time periods using Fisher’s exact and chi-squared tests, as appropriate. Multivariable linear regression analyses compared ΔOS, ΔPFS, and ΔRR between the time periods, while controlling for specific variables. Variables chosen to include in the multivariable model were those variables that showed a statistically significant association in the univariable analysis. For all analyses, the statistical significance level was set at 0.05. All statistical analyses were conducted using R 3.6.0 and SPSS 25 (IBM).

## 3. Results

**Figure 1:**
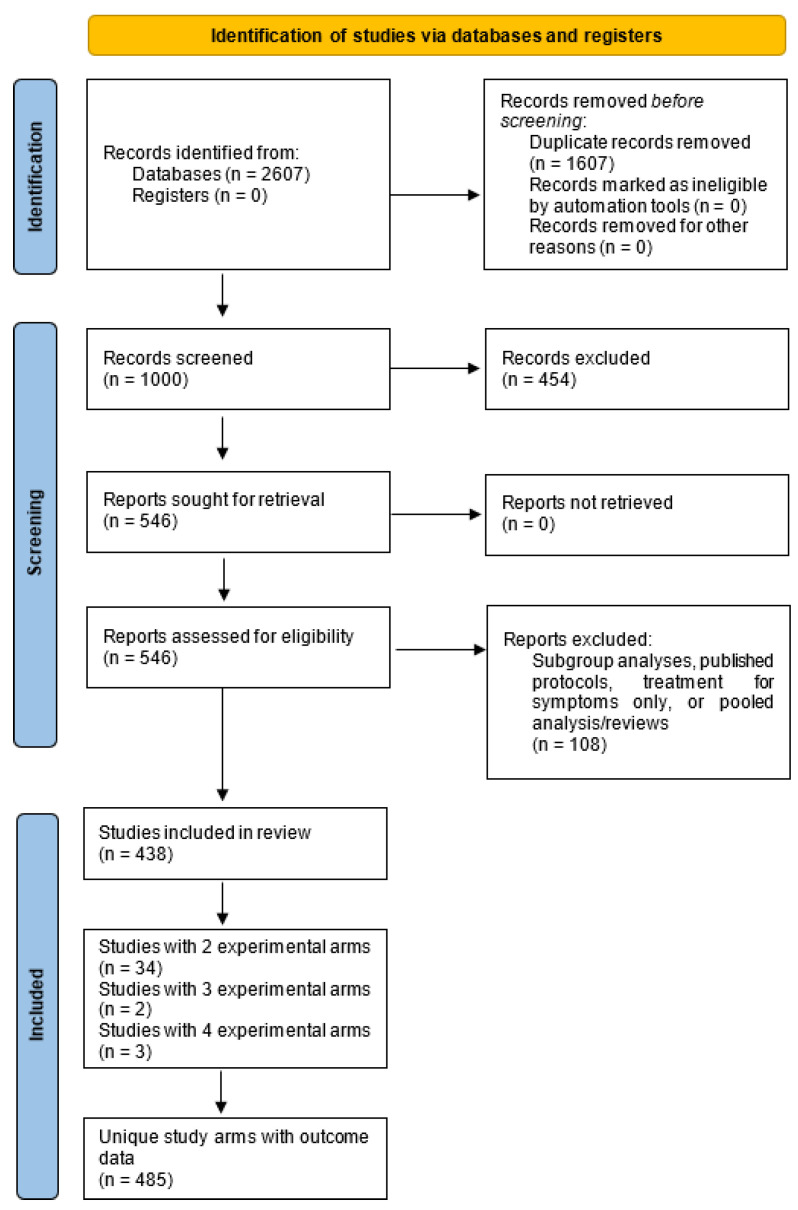
PRISMA 2020 flow diagram for article inclusion [446].

The search strategy yielded 1000 publications after duplicate removal. After screening titles and abstracts, 454 articles were excluded (Figure 1). The remaining full-text articles were screened and 108 were excluded, as they were either subgroup analyses, published protocols, treatment for symptoms only, or pooled analyses or reviews. Of the final 438 publications included [8,9,10,11,12,13,14,15,16,17,18,19,20,21,22,23,24,25,26,27,28,29,30,31,32,33,34,35,36,37,38,39,40,41,42,43,44,45,46,47,48,49,50,51,52,53,54,55,56,57,58,59,60,61,62,63,64,65,66,67,68,69,70,71,72,73,74,75,76,77,78,79,80,81,82,83,84,85,86,87,88,89,90,91,92,93,94,95,96,97,98,99,100,101,102,103,104,105,106,107,108,109,110,111,112,113,114,115,116,117,118,119,120,121,122,123,124,125,126,127,128,129,130,131,132,133,134,135,136,137,138,139,140,141,142,143,144,145,146,147,148,149,150,151,152,153,154,155,156,157,158,159,160,161,162,163,164,165,166,167,168,169,170,171,172,173,174,175,176,177,178,179,180,181,182,183,184,185,186,187,188,189,190,191,192,193,194,195,196,197,198,199,200,201,202,203,204,205,206,207,208,209,210,211,212,213,214,215,216,217,218,219,220,221,222,223,224,225,226,227,228,229,230,231,232,233,234,235,236,237,238,239,240,241,242,243,244,245,246,247,248,249,250,251,252,253,254,255,256,257,258,259,260,261,262,263,264,265,266,267,268,269,270,271,272,273,274,275,276,277,278,279,280,281,282,283,284,285,286,287,288,289,290,291,292,293,294,295,296,297,298,299,300,301,302,303,304,305,306,307,308,309,310,311,312,313,314,315,316,317,318,319,320,321,322,323,324,325,326,327,328,329,330,331,332,333,334,335,336,337,338,339,340,341,342,343,344,345,346,347,348,349,350,351,352,353,354,355,356,357,358,359,360,361,362,363,364,365,366,367,368,369,370,371,372,373,374,375,376,377,378,379,380,381,382,383,384,385,386,387,388,389,390,391,392,393,394,395,396,397,398,399,400,401,402,403,404,405,406,407,408,409,410,411,412,413,414,415,416,417,418,419,420,421,422,423,424,425,426,427,428,429,430,431,432,433,434,435,436,437,438,439,440,441,442,443,444,445], there were 34 that reported 2 experimental arms, 2 that reported 3 experimental arms, and 3 that reported 4 experimental arms. Therefore, a total of 485 unique study arms were included in the analysis. Of the 485 studies, 60 (12%) were PRE and 425 (88%) were POST. There was an even split between phase II and III studies (253 (52%) and 225 (46%), respectively). A total of 64 studies (13%) were secondary analyses, i.e., reporting updated data for a separate outcome after publication of the initial results. Furthermore, 316 (65%) of studies were in the palliative setting and 325 (67%) evaluated combination therapies. Out of the 29 different tumour types identified, the most commonly studied malignancies were breast (19%), ovarian (15%), non-small cell lung cancer (14%), colorectal (7.8%), and head and neck (7.6%).

The most common primary endpoints reported were progression-free survival, overall survival, and response rate. The differences in study characteristics pre- and post-value frameworks are summarized in Table 1. The number of studies reporting progression-free survival, overall survival, and quality of life as a primary endpoint increased marginally (45% vs. 50%, 15% vs. 21%, 3% vs. 7%, *p* = 0.011, respectively). There was a decrease in use of response rate as the primary outcome POST (27% vs. 12%, *p* = 0.011). In the POST-value frameworks era, there were significantly more studies examining novel therapies, such as targeted therapies (increased from 15 to 17%, *p* = 0.007) and immunotherapies (increased from 2 to 17%, *p* = 0.007), while fewer studies examined cytotoxic therapy agents in the POST era (decreased from 35 to 26%, *p* = 0.007). Trials performed in the first-line setting increased (45 to 48%, *p* < 0.001), while trials performed in subsequent lines decreased during the POST-value framework era (53 to 34%, *p* < 0.001). Trials that were secondary analyses more commonly reported QOL as the primary endpoint (32/64, 50%) compared with overall survival (18/64, 28%) or progression-free survival (12/64, 19%), *p* < 0.001. There was a significant increase in the number of studies evaluating palliative therapy POST (42% vs. 69%, *p* ≤ 0.001). There were no significant improvements in trials reporting or demonstrating improvement in QOL.

There were 232 articles that reported OS results, 284 for PFS, and 198 trials that reported both OS and PFS results. The results of the univariable analyses are outlined in Table 2. There was a statistically significant, modest improvement in median ΔOS in the POST era (*n* = 232 evaluable studies, 1.2 vs. −0.2 months, Wilcoxon *p* = 0.006). Similarly, median ΔPFS increased in the POST era (*n* = 284 evaluable studies, 1.4 vs. 0.2 months, Wilcoxon *p* = 0.02). There was no significant difference in ΔRR in the POST era compared to the PRE era.

In the linear multivariable regression analysis, median ΔOS was 2.86 times greater in the POST era compared with the PRE era (95% CI 0.46 to 5.26, *p* = 0.02), while controlling for potential confounders (drug mechanism of action, line of therapy, disease setting, and primary endpoint). Of the potential confounders, only disease setting showed a continued positive association in the multivariable model; compared with trials conducted in the curative setting, ΔOS was 2.66 months lower in palliative trials (95% CI −4.82 to −0.51, *p* = 0.02). Similarly, median ΔPFS was 1.59 times greater in the POST era compared with the PRE era (95% CI 0.03 to 3.15, *p* = 0.046). The potential confounders that showed a positive association in the multivariable model were disease setting and small molecule kinase inhibitor-type therapy. Compared with trials conducted in the curative setting, ΔPFS was 1.65 months lower in palliative trials (95% CI −2.94 to −0.37, *p* = 0.01), and compared with drugs with alternative mechanisms of action, ΔPFS was 1.41 months greater with small molecule kinase inhibitors (95% CI 0.07 to 2.75, *p* = 0.04).

## 4. Discussion

Over the past decade, there has been substantial growth in the development and study of novel cancer therapies [447]. We report how the recognition of value-based cancer care has shifted clinical trial outcomes. Our analysis found only marginal improvements in overall and progression-free survival. Both the ASCO and ESMO frameworks designate QOL as an endpoint of high importance. In a Canadian value framework, QOL was ranked as the endpoint of highest value by patients, public members, and decision makers [448]. Despite its importance, our analysis found that only 25% of studies published after the value frameworks were developed used QOL as an endpoint. Additionally, only one third of those studies showed significant improvement in QOL.

In another study that examined trends in clinical benefit in the period from 2006–2015, there was no statistically significant increase in clinical benefit over the decade studied [3]. Although this study included a time period preceding the publication of the value frameworks, a recent study evaluated RCT outcomes in breast, colorectal, and non-small-cell lung cancers published in 7 major oncology journals between 2010 and 2020 [449]. Study authors reported a median OS improvement of approximately 3 months [449]. This finding is higher than our more modest observation of a median OS improvement of 1.2 months in the era after the development of value frameworks. Our analysis was not limited by tumour type or journal of publication, and this may explain the small differences in the observed magnitude of clinical benefit. By including all oncologic journals in our analysis, our results are less subject to the publication bias associated with high impact factor oncologic journals that publish a greater proportion of positive trials [450]. Our findings are similar, in identifying PFS as the most common primary endpoint in clinical trials, as well as the distribution of types of therapy [449].

Translating evidence-based guidelines into practice can be a lengthy 5–10 year process [451]. After publication of a clinical trial, drug approval and funding can take several years, depending on the given health care system structure [452]. After drug availability, prescriber buy-in is needed in order to implement the drug into common practice. Kumar et al. reviewed all FDA-approved oncologic therapies between 1 October 2015, and 20 March 2016, following the publication of the ASCO working group guidelines and value frameworks. They found that only 19% of the 47 FDA-approved treatments met the predefined clinically meaningful OS improvement [453]. When dealing with value considerations, we are likely not seeing the full impact of the value frameworks yet. Appropriate time is needed for clinical trialists to design trials reflecting patient and stakeholder value priorities, accrue to these trials and publish results. As a research community, we must continue to advocate for our clinical trials to meet a high value threshold for our patients.

The main limitation of this study is the inability to consider unmeasured and unknown confounders contemporaneous with the 2015 publication of the ASCO and ESMO frameworks. Inability to control for unknown confounders could make the findings seen in the POST period compared to the PRE period appear as though they were due to the introduction of the value frameworks, while some other event was the true reason for these findings. Our study did consider plausible reasons for changes in the magnitude of clinical benefit of new cancer treatments, such as type of drug and disease setting, and positive associations were found that contributed to the reason for the findings. Furthermore, due to the acceleration in publications of oncology clinical trials over the past decade, 88% of included studies were post-value frameworks and only 12% pre-value frameworks, which likely introduced bias in the comparison of time periods. However, this comparison between groups is descriptive in nature, and appropriate non-parametric tests were used as statistical measures to help mitigate bias.

## 5. Conclusions

In this study, we found that the development of oncology value frameworks had minimal impact on the results of published randomized clinical trials. Efforts to include clinically significant endpoints shown to impact value, such as QoL, in clinical trials are warranted.

## Figures and Tables

**Table 1:** Analysis of study characteristics pre- and post-publication of value frameworks.

Characteristic	Pre-Value Frameworks: 2010–2014 (*n* = 60)		Post-Value Frameworks: 2015–2020 (*n* = 425)		Total (*n* = 485)		*p*-Value
Primary endpoint ^1^							0.011 *^a^*
Overall survival	9	(14.5%)	96	(20.6%)	105	(19.8%)	
Progression-free survival	28	(45.2%)	233	(49.9%)	261	(49.3%)	
Response rate	17	(27.4%)	48	(10.3%)	65	(12.3%)	
Quality of life	2	(3.2%)	31	(6.6%)	33	(6.2%)	
Other	6	(9.7%)	59	(12.6%)	65	(12.3%)	
Quality of life							0.745 *^a^*
Not reported	45	(75%)	321	(75.5%)	366	(75.5%)	
Improved	3	(5%)	35	(8.24%)	38	(7.8%)	
Worse	1	(1.67%)	9	(2.12%)	10	(2.1%)	
Comparable	11	(18.3%)	60	(14.1%)	71	(14.6%)	
Type of therapy							0.007 *^a^*
Immunotherapy/cancer vaccines	1	(1.67%)	72	(16.9%)	73	(15.1%)	
Cytotoxic therapy	21	(35%)	109	(25.6%)	130	(26.8%)	
Other	7	(11.7%)	43	(10.1%)	50	(10.3%)	
Small molecule kinase inhibitor	22	(36.7%)	128	(30.1%)	150	(30.9%)	
Targeted monoclonal antibody	9	(15%)	73	(17.2%)	82	(16.9%)	
Line of therapy							<0.001 *^a^*
First	27	(45%)	205	(48.2%)	232	(47.8%)	
Second or more	32	(53.3%)	144	(33.9%)	176	(36.3%)	
Any	1	(1.67%)	76	(17.9%)	77	(15.9%)	
Disease setting							<0.001 *^b^*
Curative	35	(58.3%)	134	(31.5%)	169	(34.8%)	
Palliative	25	(41.7%)	291	(68.5%)	316	(65.2%)	
Trial design							0.112 *^a^*
Randomized phase 2	36	(60%)	217	(51.1%)	253	(52.2%)	
Randomized phase 3	22	(36.7%)	203	(47.8%)	225	(46.4%)	
Randomized phase 2/3	2	(3.3%)	5	(1.2%)	7	(1.4%)	
Secondary analysis							
Yes	3	(5.0%)	61	(14.4%)	64	(13.2%)	0.043 *^a^*
No	57	(95%)	364	(85.6%)	421	(86.8%)	
Combination therapy							
Yes	38	(63.3%)	287	(67.5%)	325	(67%)	0.558 *^b^*
No	22	(36.7%)	138	(32.5%)	160	(33%)	

^1^ 45 studies had more than one primary endpoint, *^a^* Fisher’s exact, *^b^* Chi squared.

**Table 2:** Univariate analysis of changes in clinical endpoints in the pre- and post-value frameworks eras.

Endpoint	Pre-Value Frameworks 2010–2014	Post-Value Frameworks 2015–2020	*p*-Value (WilCoxon)
Overall survival improvement ΔOS, months (*n* = 232)	Mean: −0.83	Mean: 1.65	0.006
	Median (range): −0.2 (−28.4–28.2)	Median (range): 1.2 (−25.6–19.4)	
Progression-free survival improvement ΔPFS, months (*n* = 284)	Mean: 0.75	Mean: 2.00	0.023
	Median (range): 0.2 (−5.99–11.2)	Median (range): 1.4 (−20.7–36.3)	
Response rate improvement, % (*n* = 242)	Mean: 6.4	Mean: 9.19	0.281
	Median (range): 5.0 (−21.0–46.0)	Median (range): 7.4 (−32.7–52.0)	

## Data Availability

The data presented in this study are openly available in FigShare at 10.6084/m9.figshare.16959661.

## Appendix A. Search Strategy

**Table A1:** Drug Therapy (Chemotherapy, Immunotherapy, etc.) for the Treatment of Solid Tumour Cancers. Search Planning Document.

Concept	Synonym
Drug Therapy	“drug therap*” [Keyword]; drug therapy [MeSH]; chemotherap* [Keyword]; immunotherap* [Keyword]; immunotherapy [MeSH]; “antineoplastic agent*” [Keyword]; antineoplastic agents [MeSH]; chemoradiotherap* [Keyword]; chemoradiotherapy [MeSH];
Solid Tumour Cancers ^1^	“solid tumour*” [Keyword]; “solid tumor*” “solid tumor* cancer” [Keyword]; “solid tumour* cancer” [Keyword]; “bladder cancer” [Keyword]; “bladder neoplasm*” [Keyword]; “bladder carcinoma” [Keyword]; “bladder tumor*” [Keyword]; “bladder tumour*” [Keyword]; urinary bladder neoplasms [MeSH]; “breast cancer” [Keyword]; “breast neoplasm*” [Keyword]; breast neoplasms [MeSH]; “breast carcinoma” [Keyword]; “breast tumor*” [Keyword]; “breast tumour*” [Keyword]; “cervical cancer” [Keyword]; “cervical neoplasm*” [Keyword]; “cervical carcinoma” [Keyword]; “cervical tumor*” [Keyword]; “cervical tumour*” [Keyword]; uterine cervical neoplasms [MeSH]; “colon cancer” [Keyword]; “colon neoplasm*” [Keyword]; “colon carcinoma” [Keyword]; “colon tumor*” [Keyword]; “colon tumour*” [Keyword]; colonic neoplasms [MeSH]; “colorectal cancer” [Keyword]; “colorectal neoplasm*” [Keyword]; colorectal neoplasms [MeSH]; “colorectal carcinoma” [Keyword]; “colorectal tumor*” [Keyword]; “colorectal tumour*” [Keyword]; “rectal cancer” [Keyword]; “rectal neoplasm*” [Keyword]; rectal neoplasms [MeSH]; “rectal carcinoma” [Keyword]; “rectal tumor*” [Keyword]; “rectal tumour*” [Keyword]; “endometrial cancer” [Keyword]; “endometrial neoplasm*” [Keyword]; “endometrial carcinoma” [Keyword]; “endometrial tumor*” [Keyword]; “endometrial tumour*” [Keyword]; endometrial neoplasms [MeSH]; “kidney cancer” [Keyword]; “kidney neoplasm*” [Keyword]; kidney neoplasms [MeSH]; “kidney carcinoma” [Keyword]; “kidney tumor*” [Keyword]; “kidney tumour*” [Keyword]; “lip cancer” [Keyword]; “lip neoplasm*” [Keyword]; lip neoplasms [MeSH]; “lip carcinoma” [Keyword]; “lip tumor*” [Keyword]; “lip tumour*” [Keyword]; “oral cancer” [Keyword]; “oral neoplasm*” [Keyword]; mouth neoplasms [MeSH]; “oral carcinoma” [Keyword]; “oral tumor*” [Keyword]; “oral tumour*” [Keyword]; “head and neck cancer” [Keyword]; “head and neck neoplasm*” [Keyword]; head and neck neoplasms [MESH]; “head and neck carcinoma” [Keyword]; “head and neck tumor*” [Keyword]; “head and neck tumour*” [Keyword]; “liver cancer” [Keyword]; “liver neoplasm*” [Keyword]; liver neoplasms [MeSH]; “liver carcinoma” [Keyword]; “liver tumour*” [Keyword]; “liver tumor*” [Keyword]; melanoma [Keyword, MeSH]; mesothelioma [Keyword, MeSH]; “non-small-cell lung cancer” [Keyword]; “non-small-cell lung neoplasm*” [Keyword]; “non-small-cell lung carcinoma” [Keyword]; carcinoma, non-small-cell lung [MeSH]; ”non-small-cell lung tumor*” [Keyword]; “non-small-cell lung tumour*” [Keyword]; “non-melanoma skin cancer” [Keyword]; “nonmelanoma skin cancer” [Keyword]; “nonmelanoma skin neoplasm*” [Keyword]; “non-melanoma skin neoplasm*” [Keyword]; “nonmelanoma carcinoma” [Keyword]; “non-melanoma skin cancer” [Keyword]; “nonmelanoma skin tumor*” [Keyword]; “nonmelanoma skin tumour*” [Keyword]; “non-melanoma skin tumour*” [Keyword]; “non-melanoma skin tumor*” [Keyword]; “ovarian cancer” [Keyword]; “ovarian neoplasm*” [Keyword]; ovarian neoplasms [MeSH]; “ovarian carcinoma” [Keyword]; “ovarian tumor*” [Keyword]; “ovarian tumour*” [Keyword]; “pancreatic cancer” [Keyword]; “pancreatic neoplasm*” [Keyword]; pancreatic neoplasms [MeSH]; “pancreatic carcinoma” [Keyword]; “pancreatic tumor*” [Keyword]; “pancreatic tumour*” [Keyword]; “prostate cancer” [Keyword]; “prostate neoplasm*” [Keyword]; prostatic neoplasms [MeSH]; “prostate carcinoma” [Keyword]; “prostate tumor*” [Keyword]; “prostate tumour*” [Keyword]; sarcoma [Keyword, MeSH]; “small cell lung cancer” [Keyword]; “small cell lung neoplasm*” [Keyword]; “small cell lung carcinoma” [Keyword, MeSH]; “small cell lung tumor*” [Keyword]; “small cell lung tumour*” [Keyword]; “thyroid cancer” [Keyword]; “thyroid neoplasm*” [Keyword]; thyroid neoplasms [MeSH]; “thyroid carcinoma” [Keyword]; “thyroid tumour*” [Keyword]; “thyroid tumor*” [Keyword];

^1^ Solid Tumour is quite an abstract concept, thus for consistency and inclusiveness, I have adhered to the list of solid tumor cancers from Shenandoah Oncology, https://shenandoahoncology.com/disease-drug-information/types-of-cancer/, accessed on 2 December 2020.

**Suggested Search String:** (“drug therap*” OR chemotherap* OR immunotherap* OR “antineoplastic agent*” OR chemoradiotherap*) AND (“solid tumour*” OR “solid tumor*” OR “solid tumor* cancer” OR “solid tumour* cancer” OR “bladder cancer” OR “bladder neoplasm*” OR “bladder carcinoma” OR “bladder tumor*” OR “bladder tumour*” OR “breast cancer” OR “breast neoplasm*” OR “breast carcinoma” OR “breast tumor*” OR “breast tumour*” “cervical cancer” OR “cervical neoplasm*” OR “cervical carcinoma” OR “cervical tumor*” OR “cervical tumour*” OR “colon cancer” OR “colon neoplasm*” OR “colon carcinoma” OR “colon tumor*” OR “colon tumour*” OR “colorectal cancer” OR “colorectal neoplasm*” OR “colorectal carcinoma” OR “colorectal tumor*” OR “colorectal tumour*” OR “rectal cancer” OR “rectal neoplasm*” OR “rectal carcinoma” OR “rectal tumor*” OR “rectal tumour*” OR “endometrial cancer” OR “endometrial neoplasm*” OR “endometrial carcinoma” OR “endometrial tumor*” OR “endometrial tumour*” OR “kidney cancer” OR “kidney neoplasm*” OR “kidney carcinoma” OR “kidney tumor*” OR “kidney tumour*” OR “lip cancer” OR “lip neoplasm*” OR “lip carcinoma” OR “lip tumor*” OR “lip tumour*” OR “oral cancer” OR “oral neoplasm*” OR “oral carcinoma” OR “oral tumor*” OR “oral tumour*” OR “head and neck cancer” OR “head and neck carcinoma” OR “head and neck neoplasm*” OR “head and neck tumour*” OR “head and neck tumor*” OR “liver cancer” OR “liver neoplasm*” OR “liver carcinoma” OR “liver tumor*” OR “liver tumour*” OR melanoma OR mesothelioma OR “non-small-cell lung cancer” OR “non-small-cell lung neoplasm*” OR “non-small-cell lung carcinoma” OR “non-small-cell lung tumor*” OR “non-small-cell lung tumour*” OR “nonmelanoma skin cancer” OR “non-melanoma skin cancer” OR “nonmelanoma skin neoplasm*” OR “non-melanoma skin neoplasm*” OR “nonmelanoma skin carcinoma” OR “non-melanoma skin carcinoma” OR “nonmelanoma skin tumor*” OR “non-melanoma skin tumor*” OR “nonmelanoma skin tumour*” OR “non-melanoma skin tumour*” OR “ovrian cancer” OR “ovarian neoplasm*” OR “ovarian carcinoma” OR “ovarian tumor*” OR “ovarian tumour*” OR “pancreatic cancer” OR “pancreatic neoplasm*” OR “pancreatic carcinoma” OR “pancreatic tumor*” OR “pancreatic tumour*” OR “prostate cancer” OR “prostate neoplasm*” OR “prostate carcinoma” OR “prostate tumor*” OR “prostate tumour*” OR sarcoma OR “small cell lung cancer” OR “small cell lung neoplasm*” OR “small cell lung carcinoma” OR “small cell lung tumor*” OR “small cell lung tumour*” OR “thyroid cancer” OR “thyroid neoplasm*” OR “thyroid carcinoma” OR “thyroid tumor*” OR “thyroid tumour*”)

**Databases:** MEDLINE (Ovid); Evidence-Based Medicine (EBM Reviews; PubMed; CINAHL; MEDLINE (Ebsco); EMBASE;

**Grey Literature:** Clinical Trials.gov https://www.clinicaltrials.gov/, accessed on 2 December 2020.

**Table A2:** Limits.

Language	English
Age	Open
Publication Date	2010–present
Publication Type	Phase 2/Phase 3 trials
Organism	humans
